# The Holiday Tax

**Published:** 1920-07-24

**Authors:** 


### The Holiday Tax.

### Restriction on Necessary Recreation.

We should like to add our drop to the ocean of protest which has risen throughout the country against the thoroughly callous way in which the Ministry of Transport has suddenly announced a large increase in railway fares on and after August 5. A finer gesture of contempt for public convenience could hardly be conceived, especially coming as it does from a Ministry which has already provoked sharp criticism on other grounds. It is a great irritant to the national temper, and as a serious tax on holidays it is a danger to national welfare. It is absurd, of course, to imagine that the railwaymen can be paid some £70,000,000 or £80,000,000 a year in extra wages without somebody having to pay for it. The railwaymen will doubtless rattle the money in their pockets and accept with philosophic calm any share of the unpopularity which the holiday tax may bring upon them, but the wages having been so increased they have to be paid, and the vicious circle goes merrily round the mulberry bush, which in this case is the unhappy, imprisoned general public. It is another thing, however, to dash up with the extra demand just at the moment when that harassed person conveniently dubbed the general public is endeavouring to put aside for a short interval the perpetual struggle of keeping his head above inflated prices, and to enjoy the care-free recreation which comes only once a year to so many and is so vital a need to all. The Ministry must have known the necessity months ago, and instead of giving proper warning they allow people to make all their monetary calculations and arrangements and then contrive drastically to upset the whole basis of them. Some members of this Government have proved miserable psychologists. They seem to think that if only they spring upon the public a 14s. addition to the cost of a ton of coal, or a 100 per cent. extra on holiday fares, the thing is done and all the criticism and controversy from a preliminary warning will be avoided. The simple effect is precisely the contrary. Criticism is angry, irritable, and often unreasonable in consequence, and so badly has this fare business been handled that it seems more than likely that the Government will be compelled to bow to the public demands that the impost shall be postponed. It is the only fair thing to do, even if it means more later on.

NATIONAL NERVE-STRAIN.

Our consideration of this subject, apart from the equity of the case, is concerned with the needs of national recreation from the social and psychological, as well as from the health standpoint. There has been only one year of real annual holidays since the war began. The nerve-strain of living in these unsettled days is still great, and one way of renewing the national nervous energy is by the regular chance of complete change. If all holiday concessions are to be swept away by the railway companies—who found them profitable before the war and therefore should find them profitable now—there is going to be a severe financial restriction upon annual recreation, especially for those who need it most—namely, the families—because long-distance journeys will be out of the reach of many, and the circumscribed opportunities closer at hand will be in such demand that prices at nearer holiday resorts will rise proportionately. Besides that, the great masses in some of the crowded industrial centres are not within short range of the sea. It might possibly turn attention to the countryside, but why should it be restricted to the countryside, and, if it is, how do the railways expect to gain in revenue? When a start is made in rendering even necessary holidays prohibitive, it leaves the average man little to be cheerful about so far as material things are concerned. It adds almost the last irritant, but it does not stop there, for the index-figure of the increased cost of living will go up further points, there will be the usual rush of the trade unions to catch them up, the salaried people will be still further swamped, and so the game goes on. The humble taxpayer is justified at least in expecting that a Ministry of the Government should not play its share in the business—whatever the needs of the case—as if with delight.

